# Comparison of Ultra-High-Pressure and Conventional Cold Brew Coffee at Different Roasting Degrees: Physicochemical Characteristics and Volatile and Non-Volatile Components

**DOI:** 10.3390/foods13193119

**Published:** 2024-09-29

**Authors:** Qihan Shi, Ying Xiao, Yiming Zhou, Wenxiao Tang, Feng Jiang, Xiaoli Zhou, Hongxiu Lu

**Affiliations:** 1 School of Perfume and Aroma Technology, Shanghai Institute of Technology, Shanghai 201418, China; 13916902288@163.com (Q.S.); twx0321@126.com (W.T.); zhouxlsit@163.com (X.Z.); 2 School of Food and Tourism, Shanghai Urban Construction Vocational College, Shanghai 201415, China; 3 Coffee Professional Committee, Shanghai Technician Association, Shanghai 200050, China; 18515586757@163.com; 4 Shanghai Vocational College of Agriculture and Forestry, Shanghai 201699, China; luhx@shafc.edu.cn

**Keywords:** cold brew, ultra-high-pressure (UHP), roasting degree, volatile component, non-volatile component, sensory evaluation

## Abstract

The impact of the roasting degree on ultra-high-pressure cold brew (UHP) coffee remains unclear, although it has been found that UHP technology accelerates the extraction of cold brew (CB) coffee. Therefore, this study investigated the effects of three different degrees of roasting (light, medium, and dark) on the physicochemical characteristics, volatile and non-volatile components, and sensory evaluation of UHP coffee. Orthogonal partial least-squares-discriminant analysis (OPLS-DA) and principal component analysis (PCA) were used to assess the effects of different roasting degrees. The results showed that most physicochemical characteristics, including total dissolved solids (TDSs), extraction yield (EY), total titratable acidity (TTA), total sugars (TSs), and total phenolic content (TPC), of UHP coffee were similar to those of conventional CB coffee regardless of the degree of roasting. However, the majority of physicochemical characteristics, non-volatile components, including the antioxidant capacity (measured based on DPPH and ABTS) and melanoidin, caffeine, trigonelline, and CGA contents increased significantly with an increase in roasting degree. The sensory evaluation revealed that as the roasting degree rose, the nutty flavor, astringency, bitterness, body, and aftertaste intensities increased, while floral, fruity, and sourness attributes decreased. The HS-SPME-GC/MS analysis showed that most volatile components increased from light to dark roasting. Moreover, 15 representative differential compounds, including hazelnut pyrazine, linalool, butane-2,3-dione, and 3-methylbutanal, were identified by calculating the odor-active values (OAVs), indicating that these contributed significantly to the odor. The PCA showed that the distance between the three roasting degree samples in UHP coffee was smaller than that in CB coffee. Overall, the effect of roasting degrees on UHP coffee was less than that on CB coffee, which was consistent with the results of physicochemical characteristics, volatile components, and sensory evaluation.

## 1. Introduction

Coffee is a popular brewed beverage enjoyed worldwide due to its pleasant taste and complex aroma. Cold brew (CB) coffee has become the fastest-growing hot spot in the ready-to-drink coffee market in recent years. It is predicted that global revenues in coffee will reach USD 585 billion by 2025, especially due to the appearance of specialty coffees [1]. CB coffee is typically prepared by steeping coffee grounds in cold water for 12–24 h at a temperature range of 4–15 °C. Compared to hot brew coffee, CB coffee has different physical, chemical, and sensory characteristics. Most substances in CB coffee, such as furans, pyrazines, aldehydes, and esters, have a higher content, lower acidity, and softer taste [2,3]. Although CB coffee has a unique flavor and a good market prospect, its long preparation time has limited its development. The preparation of a cup of mellow-tasting CB coffee often requires an entire day. In comparison, it only takes 2–5 min to prepare a cup of hot brew coffee, which is approximately 100 times greater than the preparation time needed for CB coffee. To address this, several researchers have explored ways to speed up the extraction process, including ultrasonic-assisted [4], stirring-assisted [5], and vacuum cyclic extraction [6] CB coffee.

Ultra-high-pressure (UHP) technology has gained wide attention in the food industry, particularly in tea and traditional Chinese medicine extraction, due to its minimal impact on nutritional value and sensory quality [7,8,9]. However, only a few recent studies [10,11,12] have applied this technology to coffee extraction. The results of these studies indicate that UHP technology can accelerate coffee extraction by substituting the process of grinding. Moreover, the results of the physicochemical and sensory evaluation of UHP coffee, at 300 MPa pressure for 20 min, showed it to be closer to CB coffee. Furthermore, the extraction yield and contents of total dissolved solids, total phenol content, trigonelline, and chlorogenic acid in UHP coffee were more susceptible to improvement than those of CB coffee. However, factors that affect the quality of coffee as a beverage, such as the degree of roasting, have not been addressed.

The degree to which coffee is roasted can have a significant impact on its quality. The roasting process can eliminate unpleasant aromas and play a crucial role in determining its color and taste [13]. Complex reactions during the roasting process, such as the Maillard reaction, caramelization, and Strecker degradation [14,15], contribute to the unique flavor of coffee. Previous research on coffee’s roast level has focused on comparing the extraction conditions, physicochemical characteristics, and sensory properties of hot and CB coffee [16,17,18]. Studies have shown that as the degree of roasting increases, the total dissolved solids (TDSs) and extraction yield (EY) of CB coffee increase, while the total titratable acidity (TTA), total sugars (TSs), total phenolic content (TPC), trigonelline, chlorogenic acid, and antioxidant activity all decrease. Under the same roasting degree, CB coffee has lower TTA, TPC, and antioxidant activity than hot brew coffee, whereas the EY and TS of CB coffee are higher than those of hot brew coffee. Thus, research [19,20,21] has shown that the roasting degree has a greater impact on CB coffee compared with hot brew coffee. Additionally, the concentrations of total volatile components have been found to increase at higher roasting degrees. Furthermore, the components contributing most of the aroma of CB coffee change as the roasting degree increases. The contents of 2-butanone and 2-butenal were higher in light-roasted CB, contributing to enhanced floral and fruity flavors. Compounds with nutty and chocolate aromas, such as 2-vinylfuran, methyl furfurylthiol, 2,5-diethylpyrazine, and furfuryl methyl sulfide, were also present at higher levels. However, there has been no clear and systematic research on the effect of different roasting degrees on UHP coffee.

In this study, we investigated the impact of different roasting degrees on the quality of coffee beans after undergoing UHP treatment. We also evaluated the impact of the pressure and the roasting degree on the non-volatile and volatile components and the sensory evaluation of UHP coffee. Understanding the impact of roasting degree on UHP coffee would offer a reference for identifying the most suitable roasting beans for UHP coffee. In addition, the findings of this study are helpful for the application of UHP technology in coffee extraction and provide a theoretical foundation for the quality control of UHP coffee.

## 2. Materials and Methods

### 2.1. Coffee Sample Preparation

Arabica coffee beans from Ethiopia were selected and purchased from Yanbei Coffee Co., Ltd. (Shanghai, China). The classification of different roasting degrees (light, medium, dark) corresponds to the Agtron color value zone (73, 62, 53). All coffee beans were required to be free of visible defects, including foreign matter and damaged beans. Then, the coffee beans were passed through a color-sorting machine (S1H18, Wesort Co., Ltd., Shenzhen, China). Each coffee bean was about 10 mm in diameter.

The UHP coffee was prepared with 15 g of ground coffee (grain size of 400–600 μm) and 210 mL of water. The bottle containing the ground coffee and water mixture was placed in a UHP unit (HHP-600, Baotou Kefa High Voltage Technology Co., Ltd., Baotou, China) and left for 20 min at 300 MPa. When the pressure of the pocket in the UHP equipment returned to atmospheric pressure, the bottle was removed and the contents filtered immediately. After filtration, the UHP coffee was stored in a 4 °C refrigerator.

The CB coffee was prepared using the same powder-to-water ratio. The bottle was stored in a 4 °C refrigerator for 12 h. After filtering, the coffee samples were stored at 4 °C.

### 2.2. Total Dissolved Solids and Extraction Yield

The total dissolved solids (TDSs) were measured using a TDS refractometer (Pal-Coffee, ATAGO, Tokyo, Japan), the surface of which was cleaned with a paper towel after each use [22].

The extraction yield (EY) represented the proportion of the extracted coffee substance to the total weight of the coffee beans, and it was calculated through Equation (1) [23]:

(1) $$EY=\left(TDS\times \frac{M_{b}}{M_{gc}}\right)\times 100\%$$

where M_b_ indicates the total weight of the extract and M_gc_ represents the quality of the ground coffee used during the extraction process.

### 2.3. Total Titratable Acidity, Total Sugars, and Total Phenolic Compounds

The methods devised by Rao et al., Wang et al., Chow et al., and Bilge et al. [13,23,24,25] were used to measure the total titratable acidity (TTA), total sugars (TS), and total phenol compounds (TPC). The TTA was measured by titrating 0.1 M NaOH in 50 mL coffee until the pH reached 6.5. The TS concentration was measured through the phenol–sulfuric acid method. The TPC concentration was measured through the Folin–Ciocalteu colorimetric method using gallic acid as the standard.

The standard curve for TS and TPC is shown in Table S1 (Supplementary Materials).

### 2.4. Antioxidant Capacity and Melanoidins

Melanoidins were measured using the method of Mori et al. [26] with slight revisions. The coffee was diluted to a concentration of 1:19 and its absorbance was determined using a microplate reader (Infinite M200PRO, Tecan laboratory equipment Co., Ltd., Shanghai, China) at 420 nm.

The antioxidant activities were measured using the methods of Dong et al. [27] and Gorecki et al. [28] based on the scavenging capability of ABTS and DPPH. The units of absorbance were mmol/L Trolox. The absorbance values were calibrated with Trolox solution (10–100 μmol/L), and the data were expressed as mmol/L Trolox.

The standard curves for melanoidins and the antioxidant capacity are shown in Table S1 (Supplementary Data).

### 2.5. Caffeine, Trigonelline, and CGAs

High-performance liquid chromatography (HPLC) (LC-20A HPLC, Shimadzu, Tokyo, Japan) was used to measure the CGA, trigonelline, and caffeine contents [29].

The chromatographic column and conditions used were the following:

Caffeine contents: WodaSilTM C-18 column (150 mm × 4.6 mm × 5 µm, Shimadzu, Tokyo, Japan). Mobile phase: 24% methanol and 76% water. Temperature: 30 °C. Flow rate: 1.0 mL/min. Injection volume: 10.0 µL. Detection wavelength: 272 nm.

Trigonelline contents: WondaCract ODS-2 column (150 mm × 4.6 mm × 5 µm, Shimadzu, Tokyo, Japan). Mobile phase: 12% methanol and 88% water. Temperature: 30 °C. Flow rate: 1.0 mL/min. Injection volume: 10.0 µL. Detection wavelength: 260 nm.

CGA contents: WondaCract ODS-2 column (150 mm × 4.6 mm × 5 µm, Shimadzu, Tokyo, Japan). Mobile phase: acetonitrile and 1% acetic acid (the ratio is 15:85 (*v*/*v*)). Temperature: 30 °C. Flow rate: 1.0 mL/min. Injection volume: 10.0 µL. Detection wavelength: 260 nm. CGAs [30] included 3-CGA (Chlorogenic acid, 3-Caffeolyquinic acid), 4-CGA (cryptochlorogenic acid, 4-Dicaffeoylquinic Acid), and 5-CGA (neochlorogenic acid, 5-O-Caffeoylquinic Acid). The total CGA represented the sum of 3-CGA, 4-CGA, and 5-CGA.

The standard curves for caffeine, trigonelline, and CGAs are shown in Table S1 (Supplementary Materials).

### 2.6. Volatile Compounds

The volatile compounds present in coffee were measured using headspace solid-phase microextraction (HS-SPME) and gas chromatography/mass spectrometry (GC–MS) (Shimadzu, TQ-80, Japan) following the method of Yu [17] with minor modifications.

Qualitative analysis: The volatile compounds were identified through the retention indices (RIs) and the NIST.17 database.

Quantitative analysis: 2-Octanol (44 μg/mL) was used as the internal standard. The remaining volatile compounds in coffee were determined based on the ratio of the peak area of the internal standard to the concentration. For the calculation of RI, a C6-C30 *n* alkane series was used.

The odorant activity value (OAV) is the ratio of the concentration of an aromatic compound to the threshold, and it can be used to evaluate the extent to which aromatic compounds contribute to the flavor. The OAV was calculated using Equation (2):

(2) $$OAV=\frac{C}{C_{t}}$$

where C represents the concentration of the aromatic compound and C_t_ indicates the threshold of the aroma compound.

### 2.7. Sensory Evaluation

As per the guidelines laid out by the Specialty Coffee Association (SCA), a group of 12 trained professionals conducted a sensory evaluation of coffee samples in a specialized room, which met the requirements of ISO 8589: 2007 [31]. All of the members involved in the cupping process had undergone a rigorous coffee quality protocol laid out by the SCA and had achieved Q Arabica grader certificates (the certificates were all within the expiration dates). The evaluation was conducted using professional cupping glasses at a temperature of 20 °C ± 3 °C. The trained members tasted each sample and rated the intensity of the smell perceived retronasally. Prior to the evaluation, all referees underwent additional training to ensure that they understood the specific meaning of the sensory vocabulary. The flavor attributes included nutty/cocoa, fruity, floral, caramel, sweetness, sourness, astringency, bitterness, flavor, body, aftertaste, and overall.

### 2.8. Statistical Analysis

All experiments were repeated three times, and the results are expressed as mean ± standard deviation. Statistical analyses, including comparisons of the concentrations of volatile and non-volatile compounds, were conducted using analysis of variance (ANOVA), with *p* < 0.05 considered statistically significant (*p <* 0.05). The data were normalized in Origin 2021. GraphPad Prism 10 was used for the construction of the heatmap. And, these normalized data were used for visual mapping. Simca 14.1 was used to perform the PCA and the OPLS-DA, which are two kinds of models for data dimension reduction. Meanwhile, OPLS-DA also used to calculate the variable importance in projection.

## 3. Results and Discussion

### 3.1. Physicochemical Characteristics

Table 1 presents the physicochemical characteristics of UHP coffee at different roast levels, including measurements of TDS, EY, TA, TPC, and TS. The TDS and EY values for UHP coffee and the control group (traditional CB coffee) were not significantly different for the same roast level. However, the TDS and EY values increased significantly (*p* < 0.05) as the roast level increased. Gorecki et al. [28] showed that the decomposition of compounds during roasting might lead to the loss of soluble solids, along with the formation of new compounds. Additionally, high-temperature roasting destroys the cellular matrix, making compounds easier to extract. This means that regardless of the extraction method used, TDS and EY values continue to increase. The UHP method quickly releases a large number of water-soluble compounds under high pressure and short holding times, thus achieving TDS and EY values similar to conventional CB coffee [32].

The value of TA and TPC in UHP coffee decreases as the roast level increases. This trend is also observed in conventional CB coffee. During the roasting process, soluble protonated acidic compounds are lost due to decomposition or synthesis reactions, leading to a decrease in TA concentration. Additionally, some organic acids, like citric acid and malic acid, present in raw beans decompose during roasting [33]. The concentration of TA in coffee is the lowest at the highest degree of roasting. The variation in TA is more obvious in UHP coffee than in conventional CB coffee due to its shorter extraction time. The influence of the roast level on the TPC of UHP coffee is greater than that of conventional CB coffee. This is due to the poor stability of phenolic substances that are sensitive to temperature changes during roasting. The increase in temperature causes rapid decomposition of phenolic substances and increases the size/number of pores between coffee bean tissue structures, leading to the rapid release of some phenolic substances, like CGAs, during the initial extraction time. However, achieving equilibrium requires a longer extraction time. This effect is more significant in light roasting than in medium and dark roasting. The TS content in UHP coffee is higher than that in conventional CB coffee. However, the TS content of coffee gradually decreases with the increase in roast level. The effect of the roasting degree on the melanoidin of UHP coffee was shown to be less than that of conventional CB coffee. The coffee bean pores during dark roasting were larger compared to those of coffee beans during light roasting, thus enhancing the release of melanoidin content in CB coffee [34].

### 3.2. Non-Volatile Components

Table 2 presents the bioactive ingredients of UHP and CB coffee at various roast levels. Regardless of the extraction method used, the concentration of three common non-volatile substances, namely caffeine, trigonelline, and the chlorogenic acid group, de-creased significantly with an increase in the roasting degree (*p* < 0.05). When compared with CB coffee, the impact of UHP coffee on chlorogenic acid (CGA) content was more responsive to increased roasting. It is evident that the influence of the roasting degree on the extraction of the non-volatile components of CB coffee was higher than that of UHP coffee.

There is no significant difference in caffeine content between UHP coffee and CB coffee. Caffeine is a heat-stable alkaloid that may lose a portion of its content during roasting [34]. Furthermore, the structure of coffee beans changes when they are roasted, with the stomata closing and inorganic gases accumulating inside of the bean. This increased pressure may force the release of a small amount of caffeine. As a result, caffeine loss may be greater at higher roasting temperatures [35].

Trigonelline is important as a precursor of flavor and aroma compounds as well as a beneficial nutritional factor [36,37]. As the coffee roasting degree increases, the content of trigonelline decreases significantly (*p* < 0.05). Therefore, the UHP extraction method is useful in rapidly extracting trigonelline in a short time, and UHP coffee possesses a similar concentration of trigonelline to conventional CB coffee under dark roasting conditions. Moreover, the CGA content in medium- and light-roasted coffee was significantly higher compared with that of dark-roasted coffee (*p* < 0.05), consistent with the findings of Trugo et al. [38]. This result was attributed to the fact that the high roasting temperatures decomposed CGA, thus causing lower extraction concentrations.

### 3.3. Antioxidant Capacity

Table 3 presents the antioxidant capacity of CB coffee after UHP treatment in terms of DPPH and ABTS free radical scavenging capacity. This study revealed that the antioxidant capacity of both UHP coffee and conventional CB coffee decreased significantly with an increase in the roasting degree (*p* < 0.05). There was a significantly higher decrease in the DPPH free radical scavenging ability of UHP coffee during medium and dark roasting, while the ABTS scavenging capacity of conventional CB coffee had a more significant fall during light to medium roasting. Furthermore, the research demonstrated that the antioxidant capacity of coffee was affected by various factors, such as the CGA content, the total phenol content, and melanoid substances. These factors determine the strength of coffee’s antioxidant [33] properties. Dark roasting had the greatest effect on these antioxidant compounds, as melanoidins and other antioxidant compounds produced during roasting were not soluble in low-temperature water, which, in turn, reduced the extraction efficiency of the cold extraction process [18]. Therefore, under dark-roasting conditions, the gap in the antioxidant capacity between UHP coffee and CB coffee was smaller. Substances that are difficult to dissolve in low-temperature water are easily dissolved at high pressures, making up for the time effect of conventional CB coffee extraction.

### 3.4. Volatile Composition

**Figure 1:**
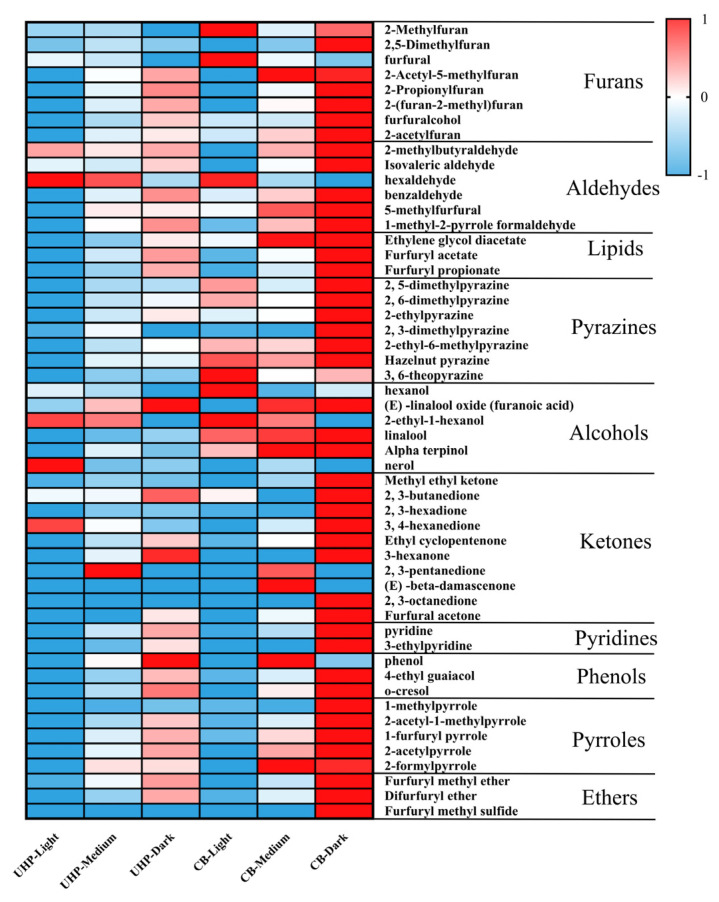
Volatile components of UHP and CB coffee at different roasting degrees. The contents of all compounds were normalized when constructing the heatmap. Red and blue colors represent the relative contents of compounds. Blue indicates lower contents, with values closer to −1, while red indicates higher contents, with values closer to 1.

Figure 1 displays heat maps of volatile compounds found in UHP and CB coffee. This study aimed to investigate the effect of different roasting degrees on the volatile components of UHP coffee, and it detected 53 volatile compounds. The most prominent compounds found were furan, pyrazine, esters, and aldehydes. In general, the overall volatile components of both UHP and CB coffee increased as the roasting degree intensified. Among them, the contents of volatile components, such as pyrazines, furans (except furfural), esters, pyrrole, pyridine, and most aldehydes, increased significantly between light and medium roasting (*p* < 0.05). Moreover, the impact of the roasting degree on UHP coffee was comparatively less than that of CB coffee in medium to dark roasting.

Furfural is a substance that is most commonly found in light roasting. It has a sweet, woody, almond, and toasted bread aroma. Unlike other furans, its volatile components decrease as the roasting degree deepens, which is consistent with previous studies [17]. This may be due to the production of furan from furfural or furfurol [36]. Additionally, aromatic compounds, such as pyrrole, are formed in coffee beans via the Maillard reaction, which occurs during dark roasting [39]. This study also found an increase in the content of pyrroles, which are described as musty, smoky, and herbal, negative flavors. It is worth noting that furfuryl acetate, which has a floral, fruity, and sweet scent, shows the most significant change among all volatile compounds. Its concentration was lowest in light roasting and highest in dark roasting. However, despite its higher content, UHP and CB coffee showed significantly lower sensory scores in floral and fruity aromas than light and medium roasting in the sensory evaluation of UHP coffee under different roasting degrees.

Pyrazine is the primary aroma-active compound found in medium- and dark-roasted coffee. It is formed through the Maillard reaction that occurs during coffee roasting [40]. The pyrazine compounds are often responsible for producing roasted and nutty odors in various heat-treated foods, such as coffee and cocoa aromas [41]. However, despite the similar proportion of pyrazine volatile components in UHP coffee and CB coffee, the nutty aroma intensity in UHP coffee is lower than that of CB, and the sensory evaluation score is also lower. Dark-roasting coffee samples contain Furol iso-valerate, ethyl guaiacol, cocoa crotonal, 2-acetylpyrrole, phenol, o-cresol, and 2-formylpyrrole, most of which provide negative odors, such as musty and smoky odors. The previous literature has also reported that guaiacol, which is detected only in dark-roasting coffee, makes an important contribution to coffee’s aroma [42]. The guaiacol content increases significantly during the roasting process, which may be responsible for the increased smoky taste of the coffee samples.

In addition, the effects of different degrees of roasting and treatments on the coffee samples were examined using OPLS-DA by calculating the VIP, which reflects the magnitude of the contribution of aromatic compounds to the overall fit and classification power of the sample. Usually, only compounds with OAV > 1 and VIP > 1 are recognized as major contributors to aroma. As shown in Table 4, 15 compounds were detected and arranged from high to low according to the magnitude of the OAV. Hazelnut pyrazine, linalool, butane-2, 3-dione, 3-methyl butanal, and furfuryl methyl sulfide, described as hazelnut, floral, sweet, and fruity, were the top five compounds contributing the most to the aroma, with all having OAVs greater than 100. Furthermore, the OAVs were higher as the degree of roasting increased. These results thus explain not only the contribution of these compounds to the coffee aroma in relation to different roasting degrees but also the difference between UHP and CB coffees.

### 3.5. Sensory Analysis

As the degree of roasting increases, the intensity of the nutty flavor, astringency, bitterness, body, and aftertaste also increases. In contrast, the intensity of the floral flavor, the fruity flavor, and the sourness decreases with the increase in the roasting degree. Additionally, medium roasting results in a higher intensity [36,40] of caramel flavor. As previous studies have also shown, CB coffee at medium roasting has a stronger caramel aroma, a more balanced flavor, and a higher overall rating than CB coffee at dark roasting. This is due to medium-roasting coffee being more integrated with other coffee-like characteristics, such as bitter and roasty flavors, while dark-roasting coffee has lower non-coffee-like characteristics, such as sweet, fruity, and coffee-like characteristics, such as bitter and roasty flavors. Moreover, at all roasting degrees, CB coffee has a higher intensity of nutty flavor, astringency, bitter flavor and aftertaste. The content of aldehydes, esters, and pyrazines in CB coffee was also higher than that in UHP coffee (Table 5). These representative volatile components may be one of the reasons for the difference in the sensory evaluation between the two different extraction methods.

### 3.6. Principal Component Analysis

**Figure 2:**
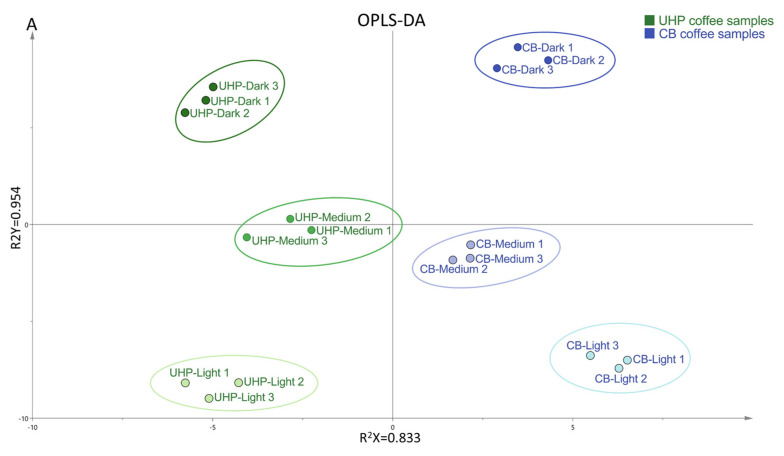

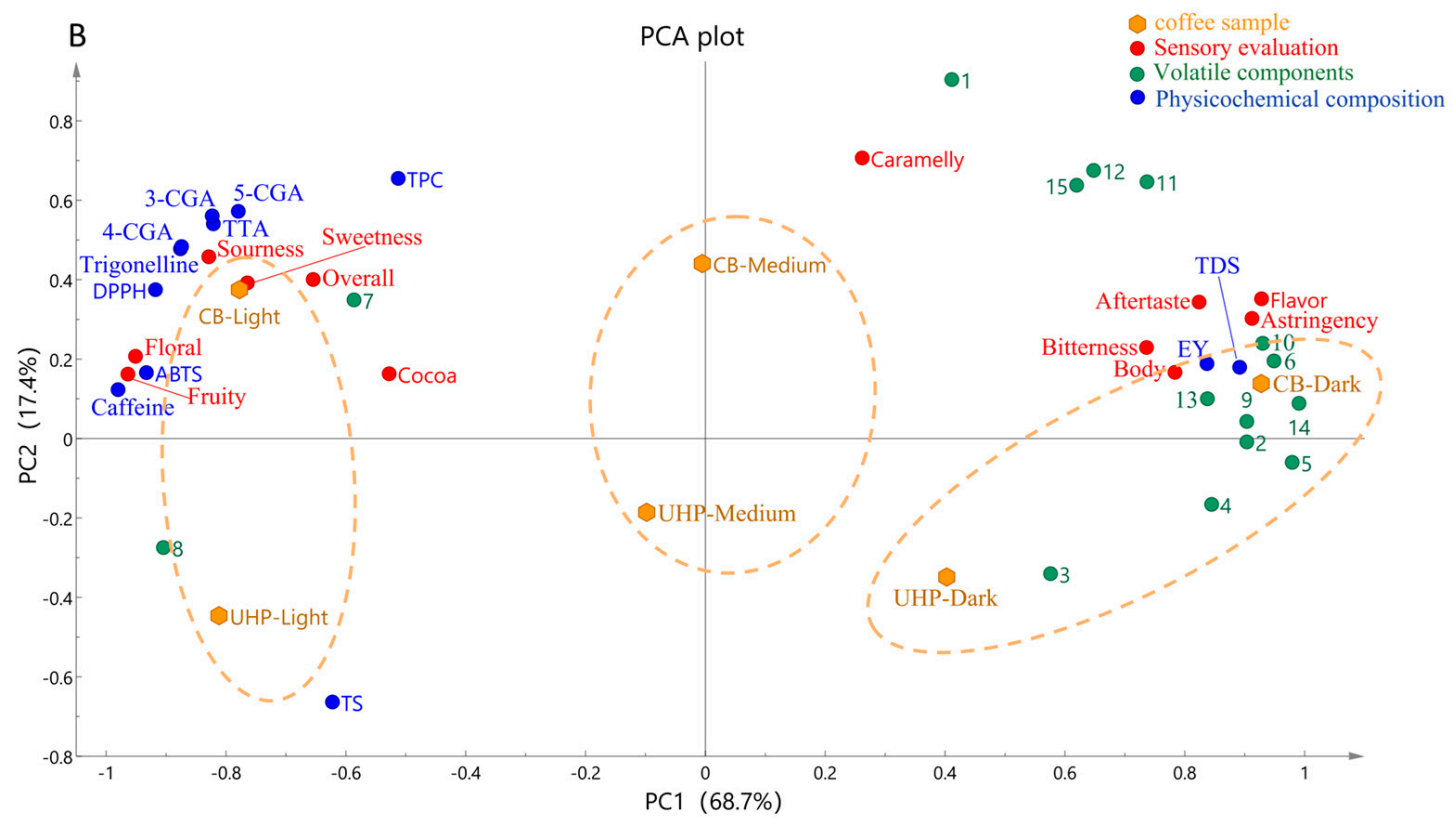
OPLS-DA plot of coffee samples (**A**) and PCA plot with coffee samples, physicochemical characteristics, sensory evaluation, and non−volatile and volatile components (**B**). The volatile components in (**B**) are the major contributors to coffee odor shown in Table 4. These compounds are listed as follows: 1. hazelnut pyrazine; 2. linalool; 3. Butane-2,3-dione; 4. 3-methylbutanal; 5. furfuryl methyl sulfide; 6. 1-Methylpyrrole-2-carboxaldehyde; 7. Hexan-1-ol; 8. hexanal; 9. 2-methylbutanal; 10. 1-Furfurylpyrrole; 11. 5-methylfurfural; 12. 2-ethyl-6-methylpyrazine; 13. 4-Ethyl-2-methoxyphenol; 14. o-cresol; 15. 2, 6-dimethylpyrazine.

Figure 2A shows an OPLS-DA plot of UHP and CB coffee samples at different roasting degrees. The six types of coffee samples are well-separated. Among them, the distance between UHP medium coffee and CB medium coffee is the closest. Figure 2B shows the PCA of physicochemical characteristics, non-volatile components, sensory evaluation, and the major contributors to the coffee’s odor. The PCA components explain variances of 68.7% and 17.4%, resulting in a total variance explained of 86.7%. CB coffee was found in the positive PC2 axis, while UHP coffee was located in the negative PC2 axis, which clearly separated the two. Additionally, there was a significant difference between the two coffees due to their different degrees of roasting. Lightly roasted coffees were distributed on the left side of the PC1 axis. Medium-roasted coffees were located near the origin, while dark-roasted coffees were on the right side of the PC1 axis. The difference between UHP and CB coffee at medium roasting was smaller compared to light- and dark-roasting coffees. This indicates that the influence of the roasting degree on UHP coffee was less than that of CB coffee. In other words, medium-roasting coffee beans are more suitable for UHP extraction. In addition, the distance between the three in UHP coffee was much smaller than in CB coffee and corresponded with the positions of the different coffee samples in the OPLS-DA plot (Figure 2A) and the magnitude of change in the OAV (Table 4).

CB coffee, when dark-roasted, is characterized by relatively richer volatile compounds. The positive flavors of pyrazines, furans, esters, and ketones, which represent the volatile substances of nuts and caramel, and the negative flavors of pyrrole, pyridine, and ethers, which represent the herbal, smoky, and plastic aroma components, are all clustered around the deep-roasted CB coffee. Although they had a higher volatile concentration, the sensory scores of the floral and fruity flavors were significantly lower compared with those of the lightly baked sample group, which is consistent with the results of previous studies [43]. Light-roasted CB coffee is characterized by a higher concentration of non-volatile components and antioxidant capacity. Regarding the sensory evaluation, they contain sour, sweet, and floral flavors, so the overall evaluation score was also higher. Light-roasted UHP coffee had a higher TS, which facilitated the release of sugar substances. Longer holding times (in the CB) have been shown to improve the retention of flavor compounds and, thus, create richer aroma components [44]. Although the UHP coffee had a lower flavor intensity than conventional CB coffee, it had similar flavor substances to CB coffee in a shorter time and a lower negative aroma, which effectively resolved the issue of long extraction times in CB coffee.

## 4. Conclusions

In this study, physicochemical and flavor characteristics were compared between UHP and CB coffee obtained using different degrees of roasting. This study found that the degree of roasting resulted in similar trends in the physiochemical indices and non-volatile component contents. Higher roasting degrees led to smaller variations in the contents of melanoidin, CGAs, and antioxidant capacity in UHP coffee relative to CB coffee.

Moreover, the sensory evaluation indicated that CB coffee has a richer nutty-like flavor, astringency, bitterness, flavor, body, and aftertaste than UHP coffee. HS-SPME-GC-MS analysis revealed that the contents contributing to odor, including aldehydes, esters, pyrazines, alcohols, pyridines, phenols, pyrroles, and ethers, in UHP coffee and CB coffee increased with the level of roasting. Compounds that showed the greatest changes in relation to roasting, such as hazelnut pyrazine, linalool, butane-2,3-dione, 3-methylbutanal, and furfuryl methyl sulfide, were found to have combined OAVs, as shown by the OPLS-DA. The PCA demonstrated that UHP coffee was more susceptible to the degree of roasting compared with CB coffee. Additionally, medium-roasted coffee beans may be more suitable for UHP coffee than light- and dark-roasted coffee beans.

The application of UHP technology in the coffee industry is yet to be fully explored. In the future, other factors that affect coffee beans, such as water quality and coffee species, should be investigated. This study also provides a basis for the application of UHP technology in the coffee field, as well as new directions for innovation in the use of CB coffee.

## Figures and Tables

**Table 1:** Impact of different levels of roasting on the physicochemical properties of coffee.

Extraction Conditions	Roast Level	Total Dissolved Solids/%	Extraction Yield/%	Titratable Acidity/(mL 0.1 mol/L NaOH)	Total Phenol Content/(mg/mL)	Total Sugar/(mg/mL)	Melanoidin/(mg/mL)
UHP (300 MPa 20 min)	Light	1.10 ± 0.02 ^c^	16.08 ± 0.25 ^c^	0.34 ± 0.01 ^ab^	4.33 ± 0.22 ^ab^	0.84 ± 0.02 ^a^	4.21 ± 0.19 ^d^
	Medium	1.18 ± 0.02 ^b^	17.11 ± 0.32 ^b^	0.32 ± 0.01 ^b^	3.40 ± 0.19 ^c^	0.81 ± 0.01 ^a^	5.23 ± 0.33 ^d^
	Dark	1.28 ± 0.03 ^a^	18.12 ± 0.38 ^a^	0.27 ± 0.01 ^c^	3.02 ± 0.13 ^d^	0.73 ± 0.02 ^b^	6.35 ± 0.28 ^a^
CB (0.1 MPa 12 h)	Light	1.11 ± 0.01 ^c^	16.10 ± 0.23 ^c^	0.37 ± 0.02 ^a^	4.82 ± 0.35 ^a^	0.73 ± 0.01 ^b^	4.33 ± 0.19 ^d^
	Medium	1.21 ± 0.02 ^b^	17.39 ± 0.29 ^b^	0.35 ± 0.01 ^ab^	4.49 ± 0.24 ^ab^	0.73 ± 0.02 ^b^	5.17 ± 0.25 ^c^
	Dark	1.30 ± 0.02 ^a^	18.31 ± 0.33 ^a^	0.30 ± 0.01 ^c^	4.23 ± 0.25 ^b^	0.70 ± 0.01 ^b^	5.92 ± 0.25 ^b^

Note: Different letters indicate statistically significant differences (*p <* 0.05) between treatments.

**Table 2:** Effects of different roast levels on non-volatile compounds under variable extraction conditions.

Extraction Conditions	Roast Level	Caffeine/(mg/mL)	Trigonelline/(mg/mL)	3-CGA/(mg/mL)	4-CGA/(mg/mL)	5-CGA/(mg/mL)	Total CGA/(mg/mL)
UHP (300 MPa 20 min)	Light	1.46 ± 0.03 ^a^	0.84 ± 0.07 ^a^	1.03 ± 0.06 ^bc^	0.48 ± 0.03 ^b^	0.42 ± 0.03 ^b^	1.93 ± 0.03 ^c^
	Medium	1.36 ± 0.04 ^b^	0.67 ± 0.03 ^c^	0.92 ± 0.04 ^c^	0.42 ± 0.02 ^c^	0.36 ± 0.02 ^c^	1.70 ± 0.03 ^d^
	Dark	1.22 ± 0.01 ^c^	0.52 ± 0.03 ^d^	0.68 ± 0.02 ^e^	0.29 ± 0.01 ^d^	0.27 ± 0.02 ^d^	1.24 ± 0.02 ^f^
CB (0.1 MPa 12 h)	Light	1.48 ± 0.02 ^a^	0.89 ± 0.05 ^a^	1.27 ± 0.04 ^a^	0.61 ± 0.02 ^a^	0.57 ± 0.03 ^a^	2.45 ± 0.06 ^a^
	Medium	1.35 ± 0.04 ^b^	0.79 ± 0.05 ^b^	1.09 ± 0.03 ^b^	0.50 ± 0.03 ^b^	0.43 ± 0.02 ^b^	2.02 ± 0.03 ^b^
	Dark	1.24 ± 0.02 ^c^	0.57 ± 0.02 ^d^	0.81 ± 0.02 ^d^	0.32 ± 0.01 ^d^	0.34 ± 0.01 ^c^	1.47 ± 0.02 ^e^

Note: Different letter indicate statistically significant differences (*p* < 0.05) between treatments.

**Table 3:** DPPH and ABTS antioxidant capacity of UHP and CB coffee.

Extraction Conditions	Roast Level	DPPH Antioxidant Capacity/(Trolox/(mmol/L))	ABTS Antioxidant Capacity/(Trolox/(mmol/L))
UHP (300 MPa 20 min)	Light	5.84 ± 0.38 ^b^	4.20 ± 0.23 ^b^
	Medium	5.57 ± 0.46 ^b^	2.64 ± 0.28 ^cd^
	Dark	4.26 ± 0.22 ^c^	1.53 ± 0.19 ^e^
CB (0.1 MPa 12 h)	Light	6.52 ± 0.32 ^a^	4.77 ± 0.35 ^a^
	Medium	5.87 ± 0.33 ^b^	2.89 ± 0.33 ^c^
	Dark	4.55 ± 0.29 ^c^	2.20 ± 0.21 ^d^

Note: Different letters indicate statistically significant differences (*p* < 0.05) between treatments.

**Table 4:** Odor thresholds and OAVs of the major contributors to the odor of coffee samples.

Compounds	Odor Description	Retention Index	Reference Retention Index	*m*/*z*	Threshold Value (ug/kg)	OAVs					
						UHP-Light	UHP-Medium	UHP-Dark	CB-Light	CB-Medium	CB-Dark
Hazelnut pyrazine	Nutty, Meaty, Roasted, Hazelnut	1489	1494	150, 135, 149	0.084	3660.26	5911.55	5913.94	8061.77	8563.89	8886.86
Linalool	Floral, Sweet, Rose, Woody, Blueberry	1048	1098	71, 93, 55	0.22	1511.96	2628.95	2746.82	746.49	3522.20	3734.97
Butane-2,3-dione	Strong, Butter, Sweet, Creamy, Pungent	613	589	41, 68	0.059	2765.71	2780.95	4069.59	1071.06	2992.19	4736.33
3-methylbutanal	Ethereal, Aldehydic, Chocolate, Peach	632	654	44, 43, 41	1.1	657.62	619.01	815.10	226.50	708.74	1221.61
Furfuryl methyl sulfide	Onion, Garlic, Pungent, Vegetable, Horseradish	984	979	81, 53, 128	0.4	251.22	584.52	774.63	187.91	496.11	1029.28
1-Methylpyrrole-2-carboxaldehyde;	Roasted, Nutty	991	971	109, 53, 80	37	8.81	27.57	35.57	14.58	32.02	45.96
Hexan-1-ol	Ethereal, Oil, Fruity, Alcoholic, Sweet	859	864	56, 43, 41	5.6	16.48	15.11	10.97	23.96	12.26	6.12
Hexanal	Fruity, Fatty, Leafy, Sweaty	807	832	44, 56, 41	5	26.21	22.15	8.12	25.00	7.82	3.70
2-methylbutanal	Cocoa, Coffee, Fermented, Alcoholic	704	695	41, 29, 57	84.3	7.66	9.00	10.95	4.92	10.76	16.48
1-Furfurylpyrrole	Plastic, Green, Fruity, Coffee, Vegetable	1128	1170	81, 147, 53	100	2.76	7.56	10.24	4.46	9.33	14.24
5-methylfurfural	Spice, Caramel, Maple	959	953	110, 53, 27	1110	3.74	6.00	5.98	5.74	7.23	7.95
2-ethyl-6-methylpyrazine	Nutty, Peanut, Musty, Corn, Raw, Earthy, Bread	1011	1005	121, 67, 39	500	1.74	2.59	2.95	3.33	3.18	4.20
4-Ethyl-2-methoxyphenol	Spicy, Smoky, Bacon, Phenolic, Clove	1257	1288	137, 152, 15	69.5	<1	1.80	4.39	<1	2.85	6.92
O-cresol	Phenolic, Medicinal, Herbal, Leathery	1048	1059	108, 79, 77	25	<1	1.96	4.64	<1	3.22	6.04
2, 6-dimethylpyrazine	Cocoa, Roasted, Nuts, Roast, Beef, Coffee	925	928	108, 42, 40	718	<1	1.00	1.08	1.21	1.10	1.44

**Table 5:** Sensory evaluation associated with different roasting degrees and extraction conditions.

Specific Attributes	UHP-L	UHP-M	UHP-D	CB-L	CB-M	CB-D
Flavor—Nutty/Cocoa	4.25 ± 0.28 ^d^	5.63 ± 0.37 ^c^	7.00 ± 0.52 ^b^	4.50 ± 0.40 ^d^	6.38 ± 0.33 ^b^	8.50 ± 0.65 ^a^
Flavor—Fruity	6.50 ± 0.40 ^b^	4.25 ± 0.20 ^c^	3.00 ± 0.18 ^d^	7.50 ± 0.39 ^a^	4.50 ± 0.52 ^c^	2.00 ± 0.15 ^c^
Flavor—Floral	4.50 ± 0.30 ^a^	2.80 ± 0.20 ^b^	1.00 ± 0.22 ^c^	4.50 ± 0.38 ^a^	2.50 ± 0.25 ^b^	1.00 ± 0.10 ^c^
Flavor—Caramelly	4.00 ± 0.22 ^c^	5.25 ± 0.35 ^b^	5.00 ± 0.30 ^b^	5.00 ± 0.42 ^b^	6.50 ± 0.48 ^a^	5.00 ± 0.33 ^b^
Sweetness	4.00 ± 0.32 ^b^	5.20 ± 0.29 ^a^	3.00 ± 0.20 ^c^	5.00 ± 0.43 ^a^	5.25 ± 0.37 ^a^	2.00 ± 0.25 ^d^
Sourness	5.50 ± 0.33 ^a^	4.20 ± 0.45 ^b^	3.00 ± 0.22 ^c^	5.75 ± 0.40 ^a^	4.50 ± 0.39 ^b^	2.50 ± 0.20 ^d^
Astringency	2.00 ± 0.15 ^e^	3.13 ± 0.28 ^d^	5.00 ± 0.35 ^b^	3.00 ± 0.20 ^d^	4.75 ± 0.38 ^c^	6.50 ± 0.42 ^a^
Bitterness	2.00 ± 0.10 ^e^	3.35 ± 0.30 ^d^	5.50 ± 0.40 ^b^	3.00 ± 0.28 ^d^	4.30 ± 0.42 ^c^	6.50 ± 0.42 ^a^
Flavor	4.00 ± 0.35 ^d^	5.33 ± 0.45 ^c^	7.00 ± 0.60 ^ab^	5.00 ± 0.47 ^c^	6.85 ± 0.50 ^b^	8.00 ± 0.65 ^a^
Body	3.50 ± 0.20 ^d^	4.50 ± 0.42 ^c^	7.00 ± 0.50 ^a^	5.00 ± 0.44 ^bc^	5.50 ± 0.30 ^b^	7.50 ± 0.62 ^a^
Aftertaste	4.00 ± 0.33 ^d^	5.50 ± 0.39 ^c^	7.00 ± 0.50 ^a^	5.50 ± 0.44 ^bc^	6.50 ± 0.35 ^b^	7.50 ± 0.40 ^a^
Overall	7.00 ± 0.35 ^b^	6.50 ± 0.42 ^b^	5.00 ± 0.33 ^c^	8.00 ± 0.47 ^a^	7.80 ± 0.67 ^a^	4.00 ± 0.38 ^d^

Note: UHP-L: UHP coffee at light roasting; UHP-M: UHP coffee at medium roasting; UHP-D: UHP coffee at dark roasting; CB-L: CB coffee at light roasting; CB-M: coffee at medium roasting; CB-D: CB coffee at dark roasting. Different letters indicate statistically significant differences (*p* < 0.05) between treatments.

## Data Availability

The original contributions presented in the study are included in the article/Supplementary Material, further inquiries can be directed to the corresponding author.

## Supplementary Materials

The following supporting information can be downloaded at: https://www.mdpi.com/article/10.3390/foods13193119/s1, Table S1: The standard curve.
