# Supplementary material for: Comparison of Ultra-High-Pressure and Conventional Cold Brew Coffee at Different Roasting Degrees: Physicochemical Characteristics and Volatile and Non-Volatile Components
Source: Foods. 2024 Sep 29;13(19):3119. doi: 10.3390/foods13193119 (PMC11475540; doi:10.3390/foods13193119)
Supplement: Supplementary file 1 [file foods-13-03119-s001.zip › foods-3214148-supplementary.pdf]

Supplementary Material

Content

Table S1 The standard curve

Compounds	standard curve	R^2
Total sugars	$y = 2.975x + 0.487$	0.9907
Total phenolic	$y = 10.801x + 0.273$	0.9940
Melanoidins	$y = 6.190x + 0.415$	0.9961
ABTS	$y = 1.895x - 1.042$	0.9944
DPPH	$y = 0.553x - 0.296$	0.9928
Trigonelline	$y = 1.180x + 0.125$	0.9964
Caffeine	$y = 2.845x - 0.459$	0.9936
3-CGA	$y = 5.006x - 1.693$	0.9959
4-CGA	$y = 14.199x + 1.042$	0.9989
5-CGA	$y = 8.148x - 2.654$	0.9975

[1] The Retention Time (RT) of Caffeine was 10.743 min (The RT of standard was 11.156 min.). The RT of Trigonelline was 3.750 min (The RT of standard was 5.505 min.) The RT of 3-CGA was 6.229 min (The RT of standard was 6.491 min). The RT of 4-CGA was 6.984 min (The RT of standard was 7.012 min). The RT of 5-CGA was 8.840 min (The RT of standard was 8.901 min).
